# Effects of species and sex on the gut microbiome of four laboratory-reared fruit fly lines (Diptera: Tephritidae) using full-length 16S rRNA PacBio Kinnex sequencing

**DOI:** 10.1186/s12866-025-04025-0

**Published:** 2025-07-28

**Authors:** Sayaka Aoki, Mikinley Weaver, Tyler J. Simmonds, Ikkei Shikano, Scott M. Geib, Charles J. Mason

**Affiliations:** 1 Tropical Pest Genetics and Molecular Biology Research Unit, Pacific Basin Agricultural Research Center, Department of Agriculture Agricultural Research Service, 64 Nowelo St. Hilo, HI, 96720 USA; 2 Department of Plant and Environmental Protection Sciences, College of Tropical Agriculture and Human Resources, University of Hawai’i at Mānoa, 3050 Maile Way, Gilmore Hall 513, Honolulu, HI 96822 USA; 3 Oak Ridge Institute for Science and Education (ORISE), Oak Ridge, TN 37831 USA

**Keywords:** Bacteria, Digestion, Diptera, Insect, Invasive, Pest, Symbiont

## Abstract

**Background:**

Insect gut microbiomes, including tephritid fruit flies, are shaped by multiple endogenous and environmental factors. While host species is a well-known driver of the gut microbiome of adult tephritids, the influence of sex is less clear. Our study evaluated the impacts of host sex and species influence the microbiome in laboratory-reared tephritids when controlled for location, time, and adult diet. We evaluated the gut microbiome of four lines of pest tephritid fruit fly adults (*Bactrocera dorsalis*,* Bactrocera latifrons*,* Ceratitis capitata*,* Zeugodacus cucurbitae*) using near full-length 16S rRNA sequencing with a PacBio Kinnex concatenation-based approach. We analyzed groups of males and females from each species at the same set of time, across four timepoints in a core insectary.

**Results:**

Results demonstrate a clear impact of fruit fly species on the gut microbiome composition of the different fruit flies. Furthermore, for *B. dorsalis*,* B. latifrons*, and *C. capitata*, we saw an influence of sex on ASV composition. However, while there was a separation of samples between the sexes for each timepoint, there was no characteristic male or female microbiome in all cases. The use of near full-length 16S rRNA sequencing did not have a marked improvement in beta-diversity interpretation over V4 subunit, with most detected taxa matching those described from other tephritids, but did allow for improved taxonomic classification at the genus level.

**Conclusions:**

Our results demonstrate that under laboratory conditions, different fruit fly species still exhibit distinct microbiomes. The impact of sex did have an impact on the gut microbiome of some species, but the magnitude of effect differed between hosts. This indicates that the sex has some impact on structuring the gut microbiome, but in a case-by-case basis. While full-length 16S rRNA sequencing affords improved classification, our study did not indicate an improvement over partial-fragments on beta-diversity metrics.

**Supplementary Information:**

The online version contains supplementary material available at 10.1186/s12866-025-04025-0.

## Introduction

Microbial contribution to insect fitness is relevant to the success of insect species, which represent the most diverse and abundant clade of animals [1–4]. Microbes play pivotal roles in promoting overall insect health, ultimately affecting the biology and physiological fitness of the host insects [4, 5]. The microbes form intimate associations with the insects by colonizing the various parts of insect bodies, including exoskeletons, digestive systems, hemolymph, and specialized cells [4, 5]. The digestive tracts of insects frequently harbor diverse bacterial assemblages, and these bacteria can provide benefits to the insects, such as provisioning vital nutrient uptake [6, 7] supplementing of metabolizing diets [8], detoxifying specialized metabolites [9], conferring resistance to natural enemies [10, 11], and protecting hosts from external environmental stressors [12–14].

Bacterial components of gut microbiomes can vary between and within species [15–17]. For instance, different host species under the same conditions can harbor substantially different bacterial communities. Furthermore, recent studies have demonstrated there can be insect species exhibit a sexual dimorphism of gut microbiomes [18–21]. For sexually mature insects, gut microbiota may influence reproductive success, either through mate selection [22, 23] or through facilitating reproductive output [22].

Tephritid fruit flies (Diptera: Tephritidae) are among the most important pests of fruits and vegetables worldwide [24]. Infestation of these flies can cause devastating economic losses to commercial crops [24, 25]. While approximately 70 species across 481 genera are considered significant agricultural pests, the most detrimental genera belong to *Anastrepha*,* Ceratitis*,* Bactrocera*,* Dacus*, and *Rhagoletis* [26]. In recent years, studies characterizing fruit fly microbiomes have gained a large amount of interest, aiming to improve the performance of flies in sterile insect technique programs and develop new control methods [26–28].

While numerous studies have addressed ecological aspects of tephritid gut microbiomes [29–31], differences in microbiomes between sexes of economically important fruit fly species still warrants further investigation. Among the studies where differences between sexes were examined, few clear compositional differences in the gut microbiome were detected [18, 32–38]. Additionally, microbiomes of different insect species maintained under similar environmental conditions can differ [39, 40], including in tephritid fruit flies [35, 41, 42]. Comparative analyses of closely related species under similar conditions could enhance accuracy and enable in-depth analyses of their associated microorganisms [42–44].

We took advantage of laboratory colonies of multiple fruit fly species to evaluate (1) differences in the adult gut microbiome between tephritid fruit fly species and (2) if there is a statistical signal of sexual dimorphism in the gut microbiome. In this study, we evaluated the gut microbiomes of four tephritid fruit fly species: melon fly (*Zeugodacus cucurbitae*), Mediterranean fruit fly (*Ceratitis capitata*), Oriental fruit fly (*Bactrocera dorsalis*), and Malaysian fruit fly (*Bactrocera latifrons*) reared under similar conditions in a core insectary. We evaluated sex-specific differences in the species at four discrete timepoints across four months. Our main hypothesis was that stronger inter-species differences would be observed, and any effects of sex would be minor relative to the effect of species.

Apart from our main objectives, another important aspect of our study was to evaluate the performance of long-read 16S rRNA sequencing methods and whether they could provide marked improvements to sequencing compared to the more frequently used V4 and V3-V4 hypervariable regions of the 1S rRNA. Other studies have demonstrated that full-length approaches can improve taxonomic classification resolution [45–49], however, full-length 16S rRNA performance for insect studies may exhibit an increased dependence on within-sample composition in addition to sequence variation [45, 47]. We used PacBio Kinnex concatenation technology, a strategy that boasts sequence outputs for similar costs to short-read amplicon strategies, for full-length library preparation, and sequenced on a PacBio Revio system.

## Methods

### Insect Sources

All four fruit fly species assessed in this study have been maintained and followed established standard operating procedures, at the USDA ARS Pacific Basin Agricultural Research Station in Hilo, HI, USA USA. These specimens have been reared by the USDA ARS for over 30 years, with intermittent introgression from wild populations. Larvae are reared on artificial diets (Supplemental Table 1) and after emergence, adults were fed a dry mixture of 3:1 sucrose: yeast hydrolysate and supplied water through a cotton wick. For our study, each fly species was maintained in separate rooms designated to each species. ~1500 individual *B. dorsalis*,* C. capitata*, and *Z. cucurbitae* were all maintained in a 40 × 40 × 40 cm mesh cages, while ~ 1000 *B. latifrons* were maintained in a 25 × 25 × 25 cm wood and plexiglass cage at any given time.

### Sample Collection and Processing

We collected samples at four sampling periods across weekly for each of the species. For each replicate, all species were collected on the same day, but the number of days after emergence differed by species. Fly age following eclosion was: six days for *B. dorsalis*, nine days for *B. latifrons*, seven days for *C. capitata*, and seven days for *Z. cucurbitae*. Five male and five female flies from each species were randomly selected from a cage at each of the four timepoints, yielding 20 per species, per sex for DNA extraction and sequencing. Flies were chilled at 4 °C and surface sterilized in ice-cold 10% bleach followed by two rinses in sterile, deionized water. Whole flies were immediately macerated in 200 µL Zymo DNA/RNA Shield (Zymo Research, CA, USA) using a sterilized micropestle and frozen at -80 °C until DNA extraction.

DNA was extracted from flies using a Zymobiomics 96 MagBead with some modifications based on the available laboratory equipment. Frozen samples were thawed and transferred into the lysis matrix. Lysis was performed using a Geno/Grinder 2010 (SPEX SamplePrep Metuchen, NJ, USA) by homogenizing 5× for 5 min at 1740 RPM (5 min rest between each step). Following lysis steps, extractions were completed using a KingFisher Flex Purification System (ThermoFisher Scientific Waltham, MA, USA) using the suggested manufacturer protocols. Extraction blanks were sequenced alongside samples and were included on each lysis matrix.

### 16 S-rRNA Amplification and Sequencing

Amplification of near full-length 16S SSU rRNA was performed according to Pacific Biosciences (Menlo Park, CA, USA) Kinnex 16 S protocols with minor modifications. PCRs were performed in 30 µL volumes consisting of 0.25 µM of primers with combinatorial indices designed as follows: 27 F (CTACACGACGCTCTTCCGATCT/barcode/AGRGTTYGATYMTGGCTCAG) and 1492R (AAGCAGTGGTATCAACGCAGAG/barcode/RGYTACCTTGTTACGACTT). 16 S was amplified using NEB Q5 Hi-Fidelity Hot Start Polymerase (New England Biolabs, Ipswich, MA, USA) with the following conditions: 98℃ for 30s; 22 cycles of 98℃ for 10s, 55℃ for 15s, 72℃ for 2 min; and final extension of 72℃ for 10 min. Amplicons were pooled and concatenated amplicons were produced following procedures detailed in the Kinnex 16 S rRNA kit (Pacific Biosciences) and were sequenced across two SMRT cells (two replicate groups on each in combination with other samples) on a Pacific Biosciences Revio sequencer. Read segmentation and demultiplexing of sequences were conducted using SMRTLink v.13 software (Pacific Biosciences).

Demultiplexed sequences were processed using a Nextflow pipeline implementing several quality control measures to produce high-quality reads (pb-16-nf V0.7 https://github.com/PacificBiosciences/HiFi-16 S-workflow). Sequences were reoriented and primers were removed using Cutadapt [50] and followed by being screened for Q ≥ 20. Sequences were then analyzed using dada2 [51] implemented in QIIME2 [52] using the pipeline default parameters except read lengths were set to fall within 1300–1500 bp. PacBio denoising algorithms implemented by dada2 were employed to generate ASVs. Taxonomic classifications of sequences were performed using Naïve Bayesian classifier implemented against the RDP 16 S rRNA database v.19 [53, 54].

### Statistical analysis

Following sequence processing, data were analyzed in R v. 4.4.1 implemented in R studio [55, 56]. For alpha- and beta-diversity analyses, we rarefied sequences to a depth of 20,000 sequences in vegan v.2.6-8 [57]. This value was our a priori decision to ensure we would include > 95% of the samples (Supplemental Material 1) and rarefaction curves (Supplemental Fig. 1) indicated that at this rarefaction depth that samples were approaching saturation. Furthermore, negative controls receiving sequences were below this value (Supplemental Material 1). We constructed Bray-Curtis dissimilarity matrices using these ASVs for evaluating community compositions. Stress values were high in non-metric dimensional ordination (2d stress > 0.25), so we performed principal coordinates analyses (PCo) with these dissimilarities. We constructed separate PCo plots for all data points and the separate species. Global PERMANOVA (vegan::adonis2) analyses was performed using species and sex as fixed effects and replicate as a strata effect. For comparing each species individually, we performed a separate analysis of similarity analysis (vegan::anosim) with the replicate as a strata effect in the model. For alpha-diversity metrics, we computed ASV richness, Simpson diversity, and Shannon diversity in vegan. ASV alpha-diversity metrics were analyzed using rstatix package v.0.7.2 [58], using species and sex as main effects with replicate as a block effect in the ANOVA model. Post-hoc analyses were performed using Tukey HSD between the species and between sex within each species.

To determine differentially abundant ASVs across species and between sexes, we used ANCOM-BC v.2.6.0 as our primary and pairwise analyses on our sample sets [59]. ANCOM-BC estimates and corrects sample-specific biases and performs a differential abundance analysis on log-transformed values [59, 60]. We performed this analysis in two stages, first to evaluate differentially abundant taxa between the fruit fly species with male and female flies pooled together, followed by differentially abundant taxa between male and female flies within each fly species. In both cases, we introduced replicate as a fixed effect in the model to allow for convergence and to account for variation introduced by the replicates. Differential abundance was performed on individual ASVs with Holm-corrected p-values < 0.05 and passing a sensitivity analysis for pseudo-counts. The sensitivity analysis is performed on samples with zeros, and those that do not pass this analysis are not implemented further [59]. The R packages phyloseq v1.48.0 and microbiome v1.26.0 were used in support of this analysis [61, 62].

In order to identify potential core or shared ASVs associated with the fruit fly microbiomes in our study, we evaluated occupancy-abundance distributions of the ASVs associated with the flies [63]. The 500 most prevalent ASVs were ranked according to their contribution to Bray-Curtis beta-diversity, with a cutoff of core ASVs by the last 5% increase in explanatory value by Bray-Curtis similarity [63]. A more stringent ‘elbow’ method using the first order differences between the curve was also employed. Both core and ASVs were plotted against a neutral model constructed using the R package ‘tyRa’ [64, 65]. Analysis was performed grouped across all flies (average relative abundance and occupancy scores) and within each individual fly species.

To determine if partial or full-length 16S rRNA sequences impacted the interpretation of our results, we extracted the V4 region of partially processed reads in dada2. We used the nucleotide sequences of the primers 515F and 806R [66, 67] to trim sequences to the sub-region. Reads ≥ Q20 were used for filtering and then reads were denoised with the dada2 PacBio algorithm. Resulting sequences were used to compute Bray-Curtis dissimilarities and correlations between full-length 16 S rRNA and V4 distance matrices were performed using Mantel test with Pearson correlation coefficients implemented in vegan.

Several other packages facilitated our analysis and data handling: ggpubr v.0.6.0 [68], ggh4x v.0.7.5 [69], ggtext v.0.1.2 [70], goeveg v.0.7.5 [71], and tidyverse v.2.0.0 [72].

## Results

### Diversity metrics differences between fly species and sexes

**Fig. 1:**
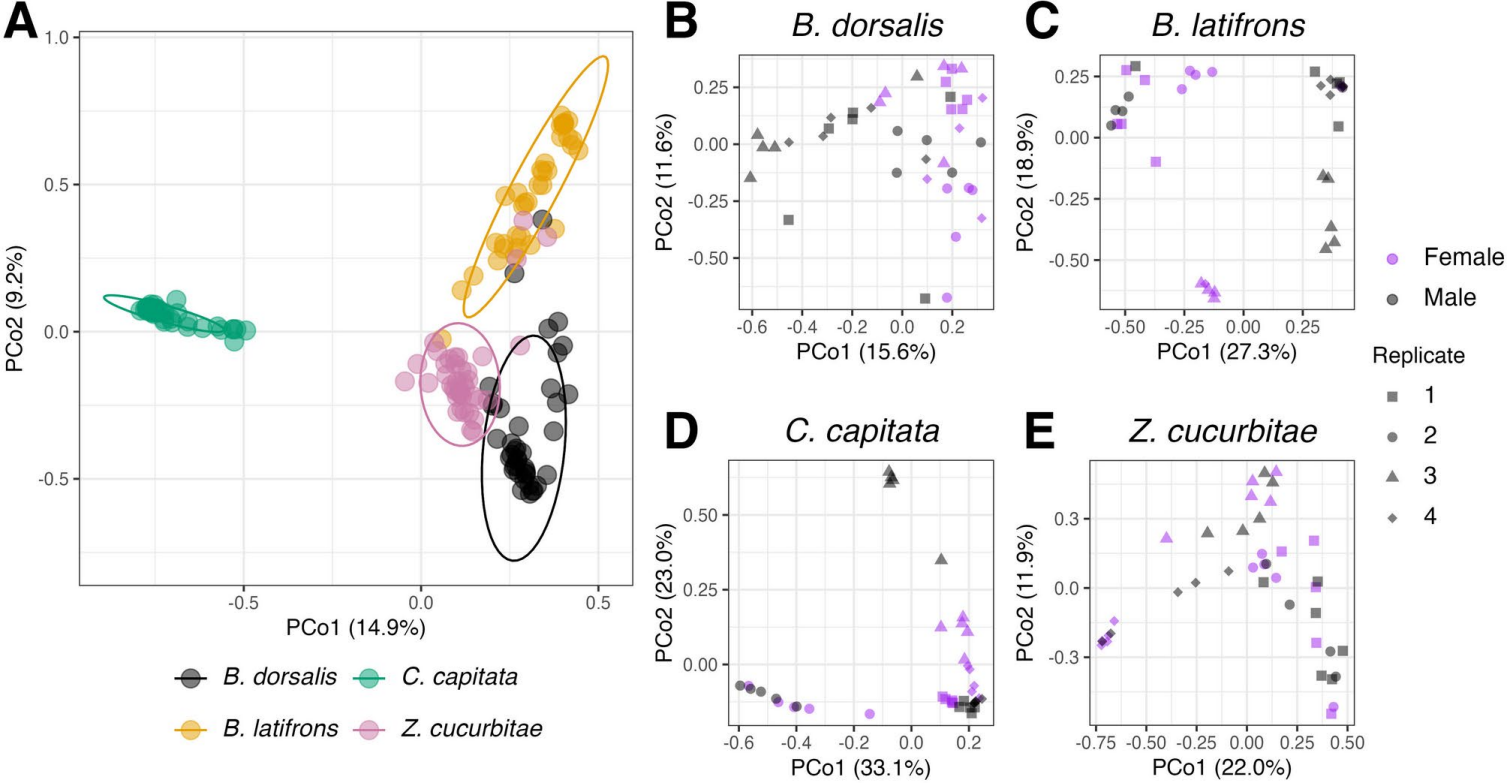
Principal coordinates analysis of fruit fly species (**A**) and of the comparisons between male and female flies of each species evaluated (**B**-**E**). Different shapes represent the cage/replicate used for each of the specimens. Ellipses in panel A represent 95% confidence intervals

We observed a clear difference between the host tephritid fruit flies in ASV composition and diversity metrics. Using Bray-Curtis dissimilarities, we observed four distinct clusters for each of the species (Fig. 1A), with *C. capitata* clustering separately from *B. dorsalis*,* B. latifrons*,* and Z. cucurbitae*. PERMANOVA analysis indicated that there were significant effects of species (F = 38.3, *p* < 0.001), sex (F = 03.43, *p* < 0.003), and the interaction between the two (F = 3.03, *p* < 0.001). The PERMANOVA model indicated that species explained far more variation (R^2^ = 0.420) than either sex (R^2^ = 0.012), or the interaction terms (R^2^ = 0.033). Because of this large effect of species and the interaction terms, we performed ANOSIM on the individual to evaluate sex-related effects within a species. We observed sex-related differences in 16S rRNA ASV compositions for *B. dorsalis* (Fig. 1B; *R* = 0.26, *p* = 0.001), *B. latifrons* (Fig. 1C; *R* = 0.22, *p* = 0.003), and *C. capitata* (Fig. 1D; *R* = 0.07, *p* < 0.01), but not for *Z. cucurbitae* (Fig. 1E; *R* = 0.01, *p* = 0.186).

**Fig. 2:**
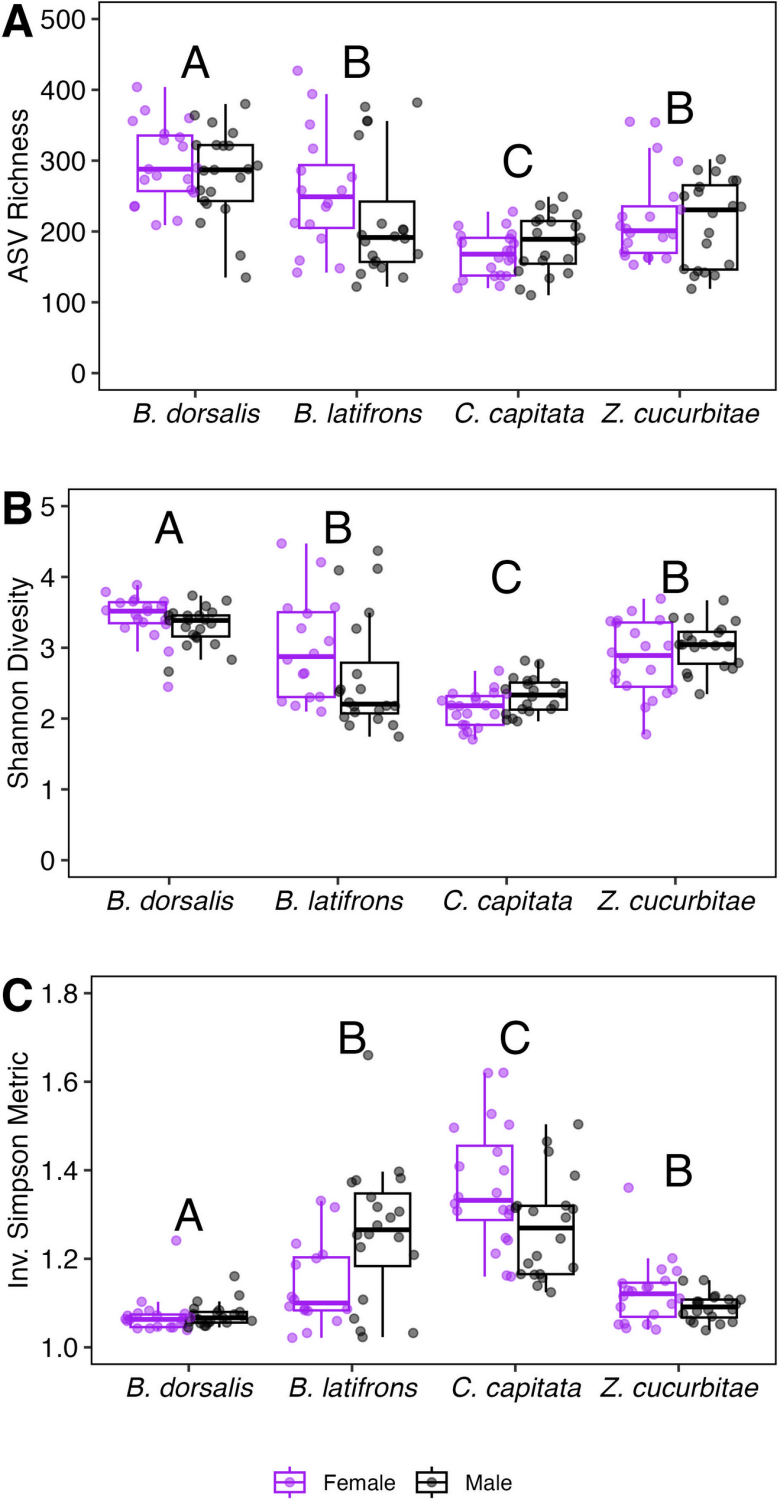
Richness (**A**), Shannon *H’* (**B**), and Inverse Simpson diversity (**C**) metrics of ASVs between different fruit fly species. Different letters denote statistically significant differences (*p* < 0.05) between different fly species. No differences were observed between sexes within a fruit fly

When evaluating ASV richness and Shannon diversity, we observed a significant effect of species, but not fly sex (Fig. 2). ASV richness was the highest for *B. dorsalis* and lowest for *C. capitata*, with *B. latifrons* and *Z. cucurbitae* in between the two (Fig. 2A; F = 34.4, *p* < 0.001). Shannon diversity (Fig. 2B; F = 40.6, *p* < 0.001) followed identical trends observed for ASV richness, but Inverse Simpson metrics followed inverse patterns (Fig. 2C; F = 54.8, *p* < 0.001).

### Taxonomic comparisons between species

**Fig. 3:**
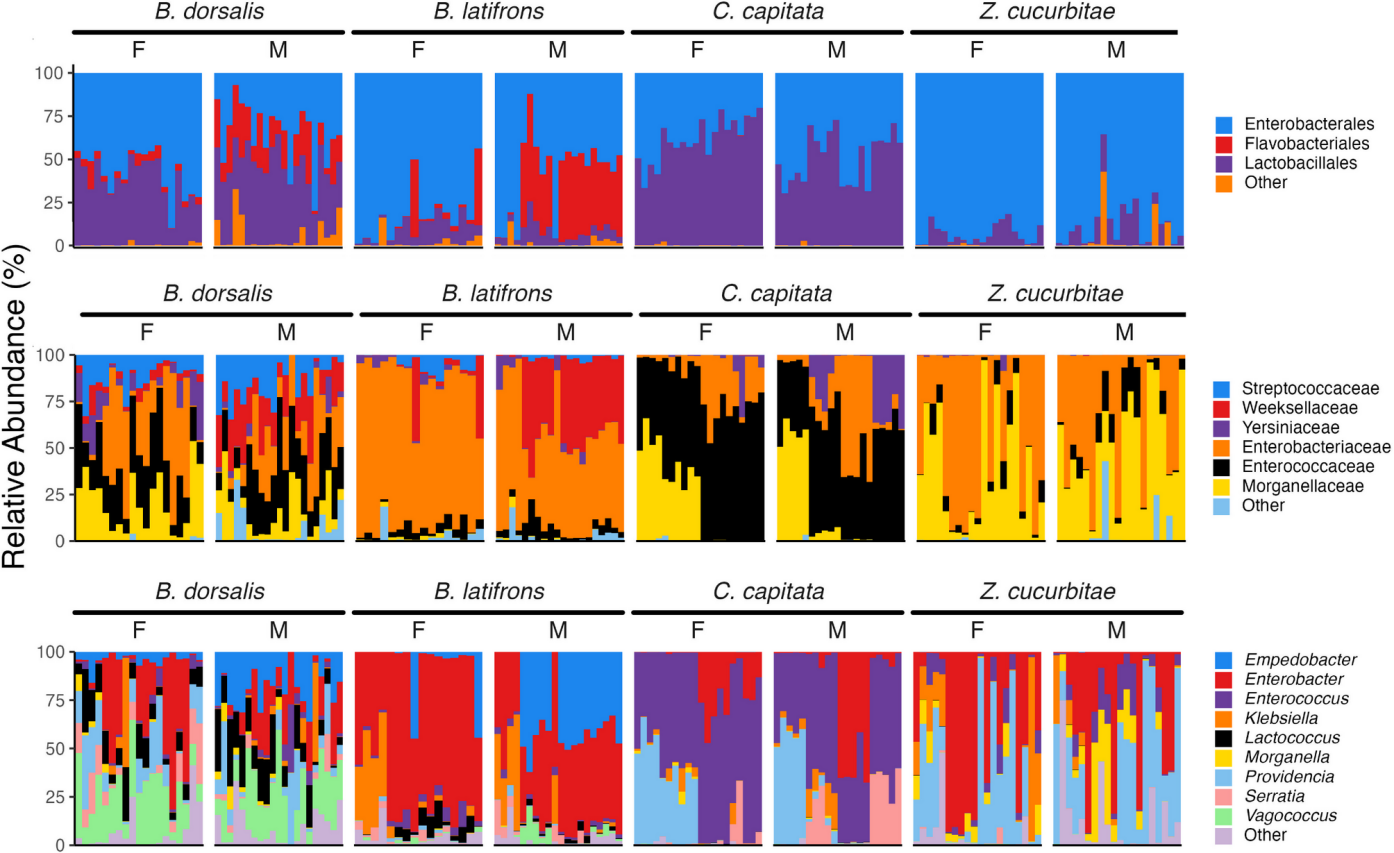
Relative abundance (%) of sequences at order- (top), family- (middle), and genus (bottom)- level classifications for four tephritid fruit fly species. ‘Other’ represents taxa comprising < 2% of the total relative abundance across all samples in each group. Each bar represents an individual fly sample. (F = Female, M = Male)

Differences in species makeup were found across the species at different levels of taxonomic classification (Fig. 3). *Bactrocera dorsalis* exhibited the highest level of taxonomic diversity compared to the other flies, and at order level were comprised mainly of Enterobacteriales and Lactobacilliales, with Flavobacteriales comprising a small portion of the communities. At genus level, *B. dorsalis* possessed greater relative abundances of *Vagococcus* sequences compared to the other flies. For *B. latifrons*, flies had high incidences of *Enterobacter*,* Klebsiella*, and *Empedobacter*. For *C. capitata*, *Providencia* and *Enterococcus* were the dominant taxa. For *Z. cucurbitae*, flies were primarily dominated by Enterobacterales, and at the genus level prominently featured *Enterobacter* and *Providencia*. At the ASV scale were some differences in their associations at the ASV level (Supplemental Figs. 2 and 3). Some ASVs were shared across species, while others had more discrete associations with certain species.

**Fig. 4:**
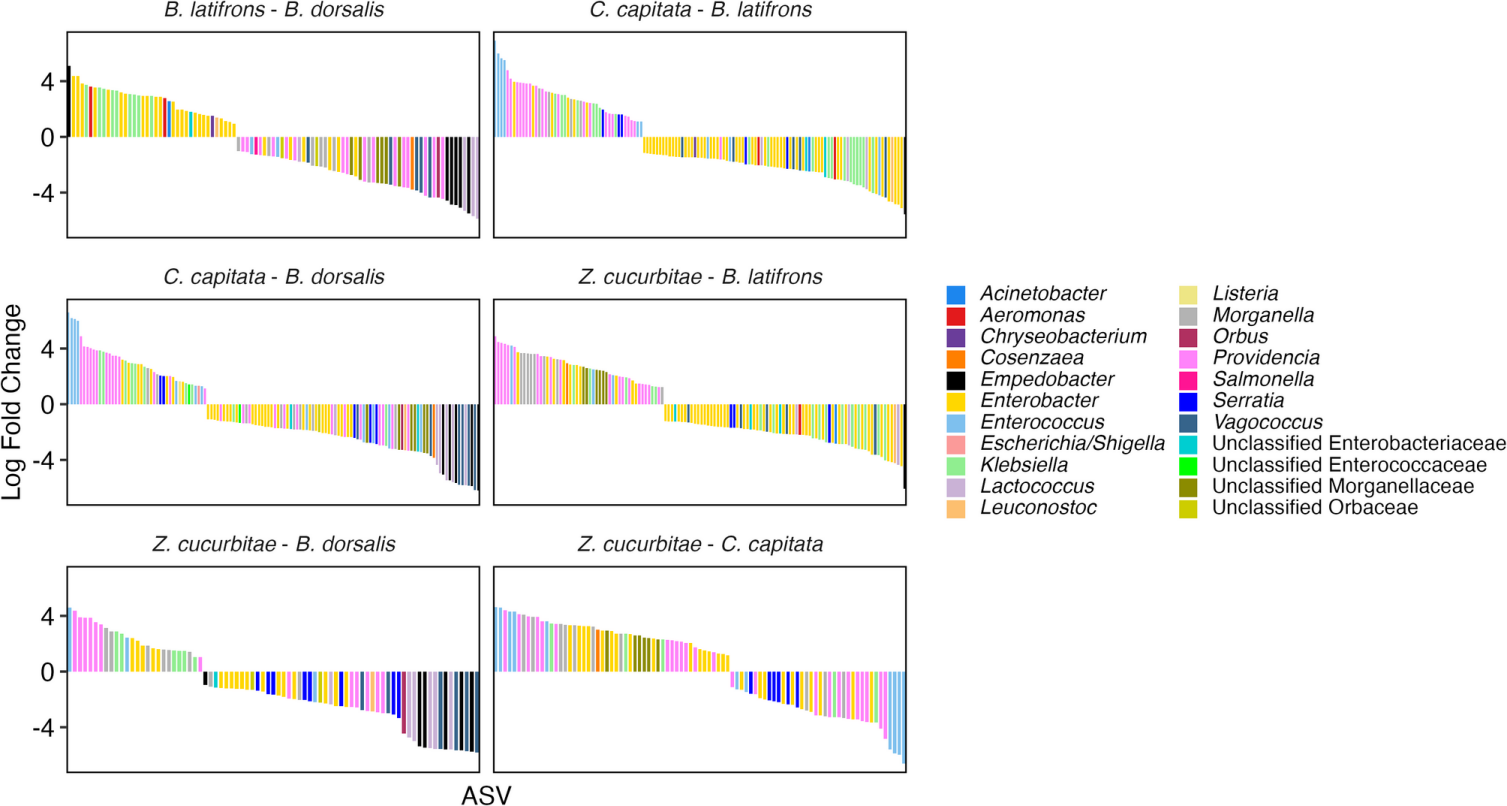
Log-fold changes of ASVs between the different host species. Only ASVs exhibiting corrected p-values < 0.05 are displayed and pseudo-count sensitivity analysis are displayed. ASV taxonomies are colored at the genus level. Positive log fold changes represent greater relative abundances first of the species comparisons, while negative log fold changes represent greater relative abundances in the second species. Models were computed using replicate/cage as a fixed block effect in the model. Full list of ASVs and associated log fold changes and ASVs are in Supplemental Material 2

Using ANCOM-BC, we identified differentially abundant ASVs between groups (Fig. 4; Supplemental Table). There were 95 differentially abundant ASVs between *B. dorsalis* and *B. latifrons*, 130 between *B. dorsalis* and *C. capitata*, 79 between *B. dorsalis* and *Z. cucurbitae*, 130 between *B. latifrons* and *C. capitata*, 126 between *B. latifrons* and *Z. cucurbitae*, and 89 between *C. capitata* and *Z. cucurbitae*. Taxonomic differences indicated some genera and families were differentially abundant, some being higher in some species over others (Fig. 4; Supplemental Material 2).

### Sex-related differences in ASVs

**Fig. 5:**
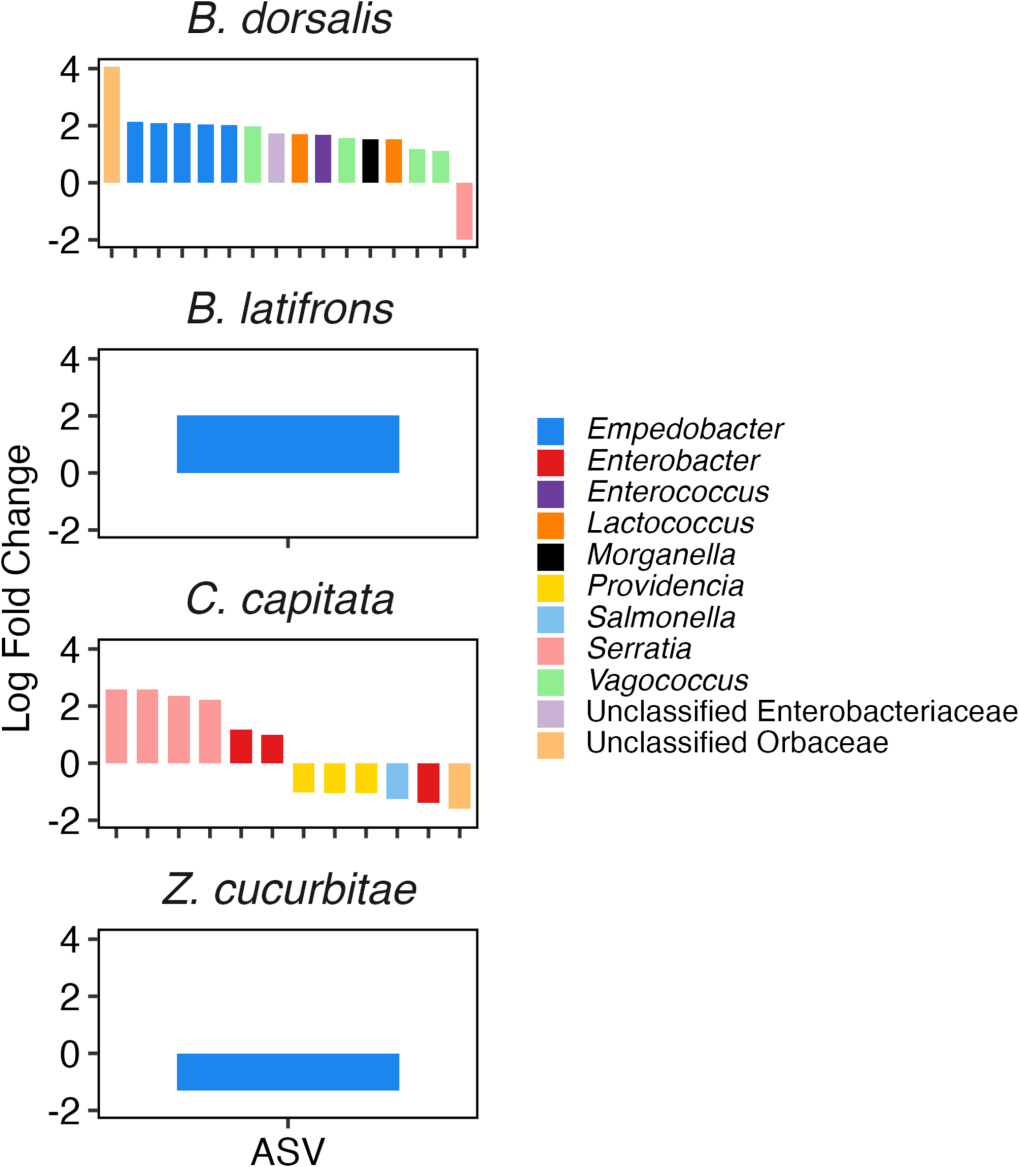
Log-fold changes of four tephritid species between male and female flies. Positive numbers represent significantly higher abundances of ASVs in males compared to females. Only ASVs exhibiting corrected p-values < 0.05 and those passing pseudo-count sensitivity analyses are displayed. Full list of ASVs and associated log fold changes and ASVs are in Supplemental Material 3

In comparison to species-related differences, there were fewer differentially abundant ASVs revealed by ANCOM-BC (Fig. 5, Supplemental Material 3). For *Z. cucurbitae* and *B. latifrons*, only one ASV was differentially abundant between males and females. For *B. latifrons*, an ASV classified as *Empedobacter* was more abundant in males than in females (Fig. 5). For *Z. cucurbitae*, a different *Empedobacter* ASV was less abundant in females than in males. For *B. dorsalis*, 16 ASVs were differentially abundant, with ASVs belong to Unclassified Orbaceae (1), *Empedobacter* (5), Unclassified Enterobacteriaceae (1), *Enterococcus* (1), *Lactococcus* (2), *Morganella* (1), and *Vagococcus* (4) being more abundant in males and a single *Serratia* being more abundant in females. For *C. capitata*, 12 ASVs exhibited significantly different abundances, with *Serratia* (4) and *Enterobacter* (2) being greater in males, and *Morganella* (3), *Salmonella* (1), *Enterobacter* (1), and Unclassified Orbaceae (1) ASVs being more abundant in females.

### Occupancy-Abundance relationships and shared ASVs between host species

**Fig. 6:**
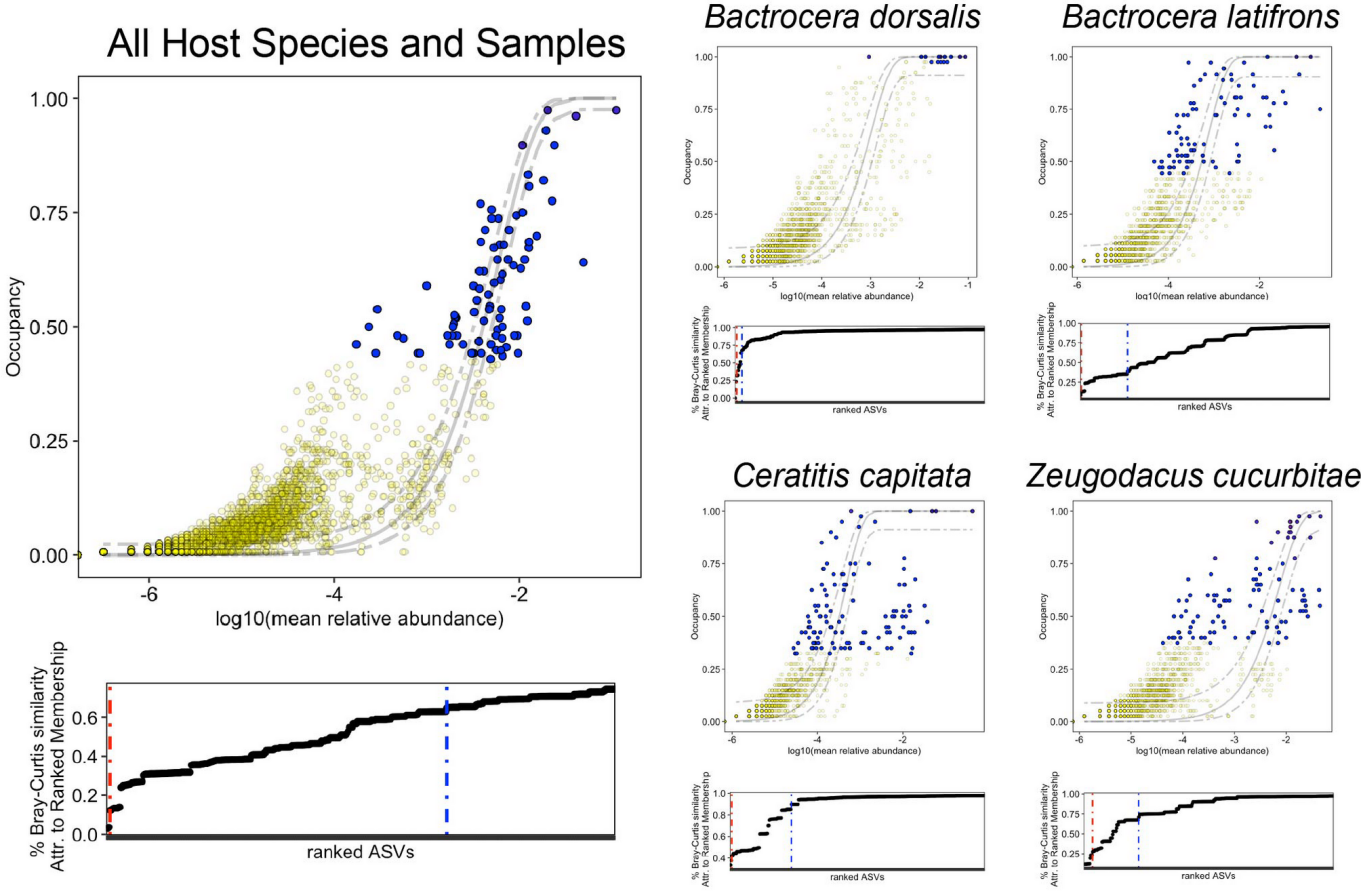
Contribution of core ASVs to Bray-Curtis dissimilarity in laboratory-reared flies using Occupancy-Abundance. Analysis incorporates the frequency of ASVs alongside their abundance to identify those that contribute to community structure. In upper graphs, blue circles represent ASVs whose rank abundance contributes ≥ 5% to the total Bray-Curtis dissimilarity (denoted by the blue line in the lower graph). More stringent ‘elbow’ using first order differences denoted by the red line on the lower graphs. Gray lines on upper graphs is the neutral model with 95% confidence intervals (models also provided in Supplemental Fig. 6). Points falling outside the confidence intervals of the neutral model can be inferred to be deterministically selected. Occupancy = 1 indicates that samples were present in all samples. Core ASVs determined with these methods are provided in Supplementary Material 3 and 4. Male and female flies were not differentiated for the analysis

We utilized occupancy-abundance relationships to infer shared and important ASVs across specimens (Fig. 6). When looking across all specimens (Fig. 6A), 84 ASVs were identified to contribute to a 5% cutoff in Bray-Curtis similarity in ranked membership. Of these, none had occupancy = 1, but 13 had occupancy > 0.75 across all fly species. We then evaluated core ASV associations within each fly species. Full list of ASVs, their occupancy, average relative abundance, and contributions to the core community are provided in Supplementary Material 4. For *B. dorsalis*, we identified 14 ASVs that had substantial contribution to the community, with nine having occupancy = 1. These included ASVs classified as *Enteroccocus*, *Vagococcus*, *Providencia*, and *Enterobacter*. For *B. latifrons*, 95 ASVs were contributing, with only two with occupancy = 1 (two *Enterobacter* ASVs). Expanding to an occupancy > 0.9 captured nine additional ASVs (*Lactococcus*,* Vagococcus*). For *C. capitata*, there were 121 ASVs, with five having occupancy = 1 and ten with occupancy > 0.9. In *C. capitata*, these included *Enterobacter*,* Enterococcus*, and *Providencia*. Finally, for *Z. cucurbitae* 110 ASVs contributed to the 5% cutoff, but none had occupancy = 1. Twelve had occupancy > 0.9, and they included taxonomic classifications of *Providencia*, *Enterococcus*, *Enterobacter*, and *Morganella*. Specific shared ASVs across fly taxa can differed in their average relative abundance by several orders of magnitude.

### Full-length 16S rRNA compared to V4 16S rRNA hypervariable region

In order to determine if there was a significant difference in full- or partial-length (V4) 16S rRNA sequences on beta-dissimilarity, we computed Bray-Curtis dissimilarities and evaluated relationships between the distance matrices using a Mantel test. There was a significant relationship between dissimilarities computed from full-length ASVs and partial (V4) ASVs (Mantel statistic *r* = 0.66, *p* < 0.001). PCo analysis corroborated those changes as V4 samples clustered similarly to those of near full-length sequences (Supplemental Fig. 4). As expected, full-length 16S rRNA did afford better taxonomic classification at the genus level compared to V4 (Supplemental Fig. 5).

## Discussion

Gut bacteria play instrumental roles in the biology of fruit fly species and at times may improve their reproductive success [15, 30]. Previous studies have demonstrated that the gut bacterial structure in tephritids could differ between species [35, 41], ontogeny [28, 73–75], and diets [76–78]. The main objective of our study was to conduct a comparative analysis of the gut microbiome in the economically significant tephritid fruit fly species under similar rearing conditions and examine whether sex impacts the microbial compositions within fly species. As expected, we observed substantial dissimilarity in the gut bacterial community compositions between fruit fly species. Additionally, we observed a statistical effect of sex on the gut microbiome. However, these effects were complex as we did not observe it for all species, and where effects were present there was not always a consistent sex-specific microbiome. Instead, there were differences between sexes at the different replicated time points. Our results further contribute to understanding factors that structure fruit fly gut microbiomes.

In line with prior observations with other tephritid fruit flies [35, 42], our analysis of ASV composition and diversity metrics demonstrated strong dissimilarities in the microbial compositions between four different host species. Among four species of our flies tested, *C. capitata* exhibited more separation from the other species surveyed. *C. capitata* had the lowest species richness and Shannon diversity metrics, but higher Inverse Simpson, indicating that out of the four species that is had the least even and most dominant community membership. Beta-diversity metrics also indicate a greater separation of *C. capitata* from the other species. One possible explanation for these observations between host taxa is differences in larval diet. Although the larval diets differ for rearing these species in the laboratory, the ingredient compositions of the diets exhibit substantial overlap (Supplemental Table 1). Larval *C. capitata* diet has almost an identical ingredient composition as *B. dorsalis* and *Z. cucurbitae* but is acidified. *Bactrocera latifrons* is also acidified but contains wheat germ and carrot as bulking agents. Overall, the carbohydrate: protein ratios differ between these species slightly, but we do not know how that affects adult microbiomes. Although microbiomes vary in composition and function across life stages in tephritids [30, 79, 80], carry-over of bacteria between developmental stages [79] may also contribute to the differences in our study. In addition to larval diet differences, phylogeny may contribute to our observed microbiome differences. All the species we examined are in the family Tephritidae, *B. latifrons*,* B. dorsalis*, and *Z. cucurbitae* are in the Dacini tribe and more closely related to each other while *C. capitata* is in the Ceratidini tribe. Phylogeny has been a relevant explanatory variable of microbiome composition between species [81], and there are likely both gut biochemical and immunological factors that drive species-specific composition.

Using an occupancy-abundance framework, we identified that there were shared ASVs across the specimens, although none were present across in all samples and few within a species. Furthermore, those that were shared between fly species were at very different abundances (Supplementary Material 4). The ASVs were also weakly associated with the neutral model (Supplemental Fig. 6), indicating that there were host-selected forces at play on composition. Overall, this suggests that the host has differential effects on associated and abundance of specific microbiota [40]. The specific ASVs that contribute to the ‘core’ microbiome of specific hosts was fairly broad, indicating that although hosts deliver greater influence on ASVs than neutral processes, there is variability across to which taxa are the most important. Others have found that combination of stochasticity and host-determinants intersect to affect the structure and distribution of taxa associated with fruit fly gut microbiomes [82, 83].

Across the four species, there was a high representation of ASVs previously detected in other tephritid systems [29, 30]. At the higher taxonomic level, Enterobacterales was the most abundant across all fly species, with the families Morganellaceae and Enterobacteriaceae being the most prevalent. Many tephritid bacterial symbionts belong to Enterobacteriaceae, notably *Enterobacter* and *Klebsiella* [22, 26, 30, 84–87]. Interestingly, while *Klebsiella* has been shown to be a dominant member of the mass-reared *C. capitata* microbiome in many facilities [27, 32, 88, 89], we detected sparse abundances here, with more ASVs classified as *Enterobacter* being detected. Within our tested colony flies, all four species had some sequences that were classified to *Providencia* and *Morganella*, which have been reported in fruit flies as both pathogens and commensal symbionts [28, 76, 90, 91]. Differences in relative abundances, and the individual ASV level (Fig. 4), suggest strain-level variation between host species.

Lactobacillales was also present in all fly species however, it was observed highly abundant in *B. dorsalis* and *C. capitata*. *Vagococcus*, a lactic acid bacteria, was previously found in *Z. cucurbitae*, *B. dorsalis*, *B. tyroni*, and *B. minax* [78, 92–95]. In our colonies, *Vagococcus* was found in *B. dosalis* and *B. latifrons* but not in the *C. capitata* and *Z. cucurbitae. Enterococcus* was most prominent in *C. capitata*, which has been observed in other studies of reared medflies although not at the relative abundances we observed [27, 96].

Flavobacteria in low frequencies in *C. capitata* and *Z. cucurbitae*, but was detected with higher abundance in male *B. latifrons* and *B. dorsalis* compared to females of the same species. *Empedobacter* (Flavobacteria) was detected in *B. dorsalis* and highly abundant in *B. latifrons*, but was absent in the other two fly species. Small percentages of Flavobacteria were previously found in *B. dorsalis* and is possibly involved in the nitrogenous waste recycling in the fly gut [18, 97, 98]. However, in other surveys of tephritid fruit flies, *Empedobacter* is relatively infrequent compared to other taxa (like those in the Enterobacteriaceae). Since *Empedobacter* is a widely distributed environmental bacteria, its detection in our study could be a result of the artificial rearing environment [98, 99].

While there were stark contrasts in the gut microbiome between species, the differences between sexes within species were more nuanced. We observed a statistically significant effect of sex on the beta-diversity of the gut microbiome of *B. dorsalis*,* B. latifrons*, and *C. capitata*. However, besides *B. latifrons*, we did not see a ‘characteristic’ male or female microbiome for *B. dorsalis* or *C. capitata*. Rather, we saw a difference in composition at each replicate timepoint and skewing composition towards one direction or another. This suggests that there is some sex-related modulation of the gut microbiome, even among co-mingling male-female populations. Some studies have demonstrated that gut microbiota can affect reproductive behavior and physiology between sexes in fruit flies [100, 101]. However, the biochemical and/or molecular mechanisms underlying the selection toward a male- or female-biased microbiomes are unclear but warrant further investigation. Understanding what drives compositional and functional differences in microbiomes between sexes would provide insight into the important linkages between digestion and reproduction in tephritid fruit flies.

A caveat of our study is that we utilized laboratory-reared fruit fly lines. Although this allows for more controlled comparisons within our study, we would be remiss not to acknowledge that wild or fruit-reared populations can harbor different microbial associates. For instance, a prior study from field populations of *B. latifrons* found high incidences of intracellular *Wolbachia* [102], which is absent from our study. *Wolbachia* can often be a pathogen, which would explain its low incidence following colony establishment. Furthermore, field populations of fruit flies scan yield more and diverse gut microbiomes [83, 103, 104]. While we presume host species, sex, maturation, and diet would be at play in contributing to microbiome composition and function, the forces that most outweigh the others still needs to be considered and evaluated.

At the very least, near full-length 16S rRNA sequences can provide more taxonomic information than markers. Similarly, in another study by our group, we found that near full-length 16S rRNA amplicons dramatically improved both taxonomy alongside beta-diversity metrics [105]. However, when selecting the V4 region in this study, we did not see a significant difference in beta-diversity metrics and interpretation of either of the main statistical effects we evaluated here. This lack of improvement in diversity metrics reflects findings in other insect species [45], which indicates that the enhanced resolution that full-length 16S rRNA amplicons affords is case-by-case.

Our study provides tangible evidence that bacterial communities are structured in part by fruit fly species. By rearing these flies in laboratory culture, we can control some of the environmental factors that influence gut microbial communities, such as adult diet. For three of our species, we observed the effects of fly sex on the gut microbiome. Improved knowledge of the mechanisms underlying the colonization and proliferation of gut microbiome members is needed. Furthermore, understanding whether the different gut microbiomes possess different or similar metagenomic content can provide insight into the functions of the different communities. Investigating these bacterial muti-species interactions and how these groups are functioning together will add valuable information to our understanding of bacterial symbiosis in tephritid flies.

## Electronic supplementary material

Below is the link to the electronic supplementary material.

Supplementary Material 1

Supplementary Material 2

Supplementary Material 3

Supplementary Material 4

Supplementary Material 5

Supplementary Material 6

## Data Availability

Desegmented, demultiplexed sequence reads have been deposited to the NCBI Sequence Read Archive under the accession number PRJNA1196954. Data have been deposited to the USDA NAL under the doi 10.15482/USDA.ADC/28898312.
