# Supplementary material for: Effects of species and sex on the gut microbiome of four laboratory-reared fruit fly lines (Diptera: Tephritidae) using full-length 16S rRNA PacBio Kinnex sequencing
Source: BMC Microbiol. 2025 Jul 28;25:455. doi: 10.1186/s12866-025-04025-0 (PMC12306104; doi:10.1186/s12866-025-04025-0)
Supplement: Supplementary file 1 — Supplementary Material 1 [file 12866_2025_4025_MOESM1_ESM.docx]

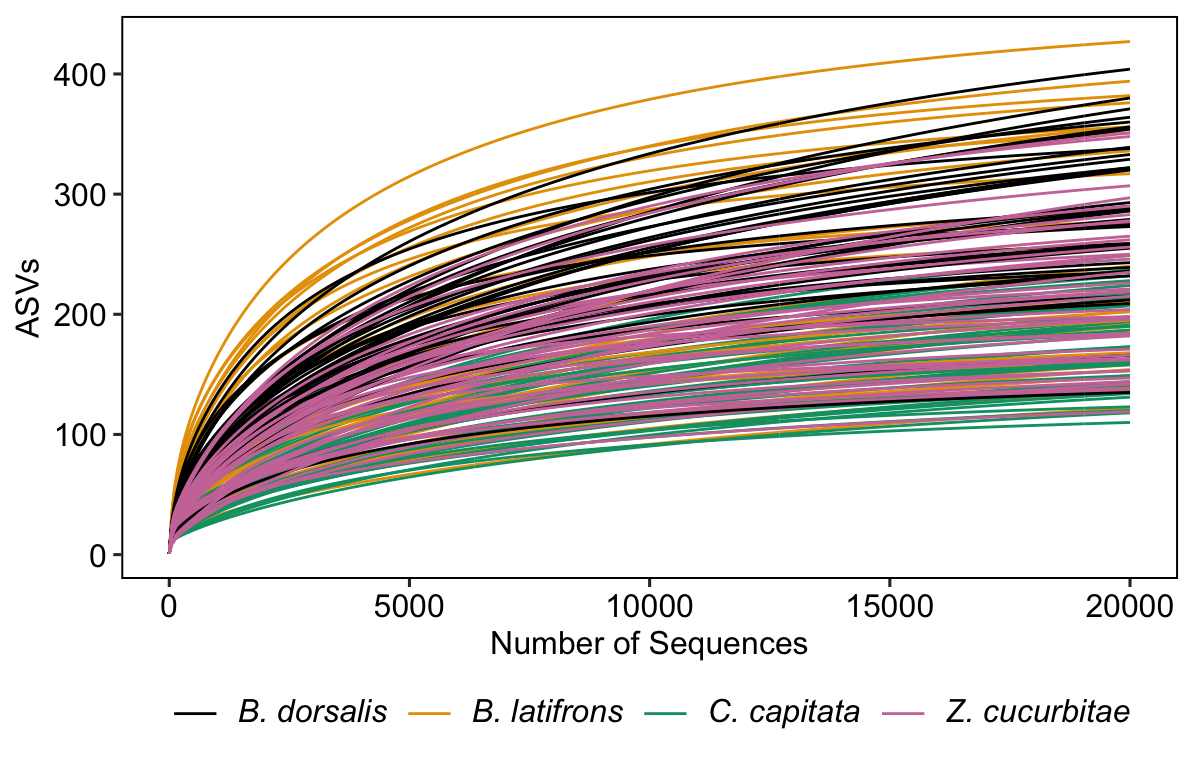


Supplemental Figure 1: Rarefaction curves of sub-sampled samples (20,000 seqs)


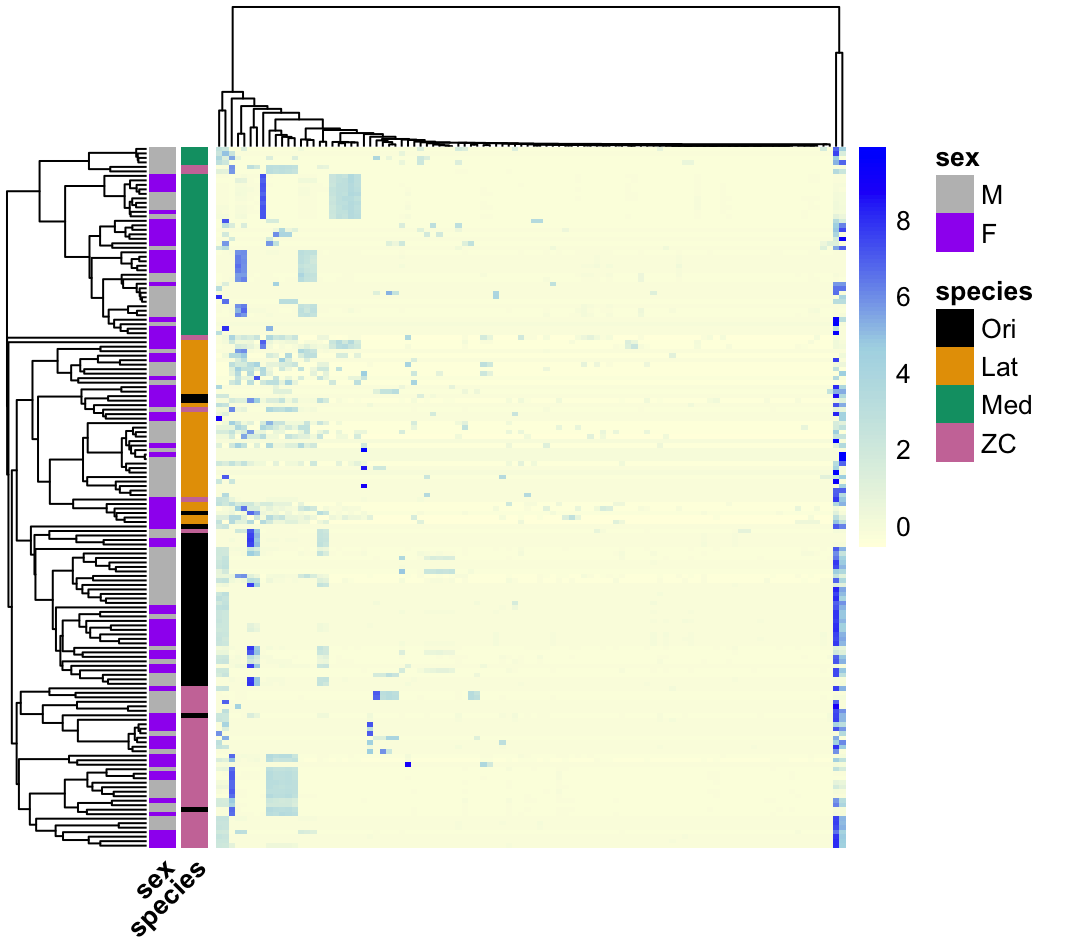


Supplemental Figure 2: Heat map of reads classified as *Providencia* (z-transformed values). Note that the abundances are relative to other *Providencia*  sequences and not the total relative abundance in the samples.


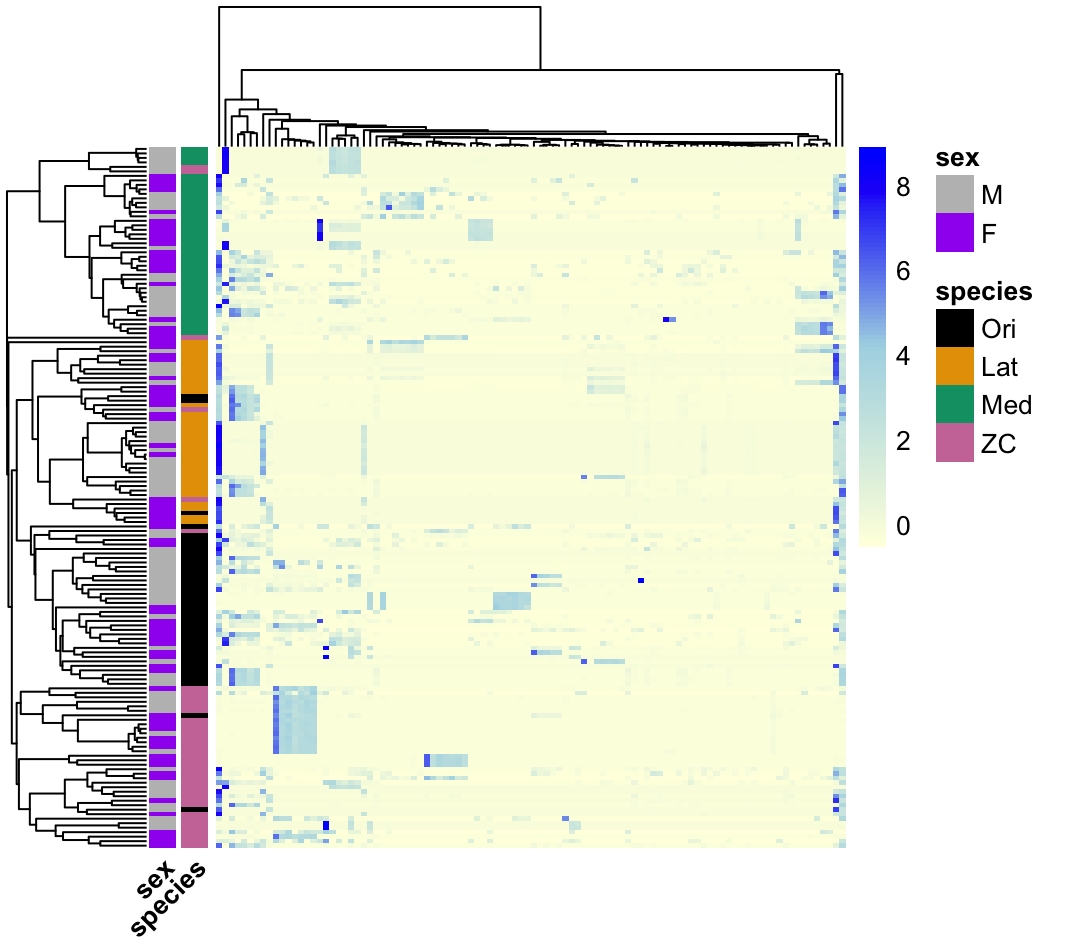


Supplemental Figure 3: Heat map of reads classified as *Enterobacter* (z-transformed values). Note that the abundances are relative to other *Enterobacter* sequences and not the total relative abundance in the samples.

Supplemental Figure 4: Principal coordinates analysis of different species using full-length (A) and V4 subunit (B).

Supplemental Figure 5: Stacked barcharts of full-length (A) and V4 subunit (B) at genus level.
