# Supplementary material for: Effects of species and sex on the gut microbiome of four laboratory-reared fruit fly lines (Diptera: Tephritidae) using full-length 16S rRNA PacBio Kinnex sequencing
Source: BMC Microbiol. 2025 Jul 28;25:455. doi: 10.1186/s12866-025-04025-0 (PMC12306104; doi:10.1186/s12866-025-04025-0)
Supplement: Supplementary file 2 — Supplementary Material 2 [file 12866_2025_4025_MOESM2_ESM.docx]

| Supplemental Table 1: Larval diet mixtures (in g) for PBARC tephritid fruit fly colonies. | | | | |
| --- | --- | --- | --- | --- |
|  | *Bactrocera dorsalis* | *Bactrocera latifrons* | *Ceratitis capitata* | *Zeugodacus cucurbitae* |
| wheat mill feed | 1585 | --- | 1548 | 1640 |
| granulated white sugar | 718 | 160 | 717 | 387 |
| torula yeast | 200 | 280 | 200 | 187 |
| nipagen | 6.7 | 17 | 12.2 | 6 |
| sodium benzoate | 5.6 | 0 | 12.2 | 6 |
| citric acid | --- | 161 | 136.05 | --- |
| wheat bran | --- | 1212 | --- | --- |
| carrot flakes | --- | 346 | --- | --- |
| water (mL) | ~2000 | ~2000 | ~2000 | ~2000 |
